# The international dimensions of antimicrobial resistance: Contextual factors shape distinct ethical challenges in South Africa, Sri Lanka and the United Kingdom

**DOI:** 10.1111/bioe.12604

**Published:** 2019-07-02

**Authors:** Eva M. Krockow, Carolyn Tarrant

**Affiliations:** ^1^ University of Leicester College of Medicine Biological Sciences and Psychology Leicester United Kingdom of Great Britain and Northern Ireland

**Keywords:** antibiotic stewardship, antibiotics, antimicrobial resistance, conflict of interests, hospitals, Rule of Rescue, veil of ignorance

## Abstract

Antimicrobial resistance (AMR) describes the evolution of treatment‐resistant pathogens, with potentially catastrophic consequences for human medicine. AMR is driven by the over‐prescription of antibiotics, and could be reduced through consideration of the ethical dimensions of the dilemma faced by doctors. This dilemma involves balancing apparently opposed interests of current and future patients, and unique contextual factors in different countries, which may modify the core dilemma. We describe three example countries with different economic backgrounds and cultures—South Africa, Sri Lanka and the United Kingdom. Then we discuss how country‐specific factors impact on the prominence of various ethical dimensions of the dilemma (visibility and moral equality of future generations; Rule of Rescue; prescribing autonomy and conflicts of interest; consensus on collective action). We conclude that a nuanced understanding of national prescribing dilemmas is critical to inform the design of effective stewardship approaches.

## 1 BACKGROUND

Antimicrobial resistance (AMR) has been characterized as one of the most serious human health threats in the 21st century, and is predicted to cost more than 10 million lives by 2050.1O'Neill, J. (2016). Tackling drug‐resistant infections globally: Final report and recommendations. *Review on Antimicrobial Resistance, 178*(23), 590. https://doi.org/10.1136/vr.i3114
 AMR refers to the evolution of drug‐resistant micro‐organisms, including bacteria, which is fuelled significantly by the overuse of antibiotics. If resistance continues to increase, common bacterial infections will eventually become untreatable with existing antibiotics.2Levy, S. B. (1998). The challenge of antibiotic resistance. *Scientific American, 278*(3), 46–53. https://doi.org/10.1038/scientificamerican0398-46; Levy, S. B. (2001). Antibiotic resistance: Consequences of inaction. *Clinical Infectious Diseases, 33*(3), 124‐129. https://doi.org/10.1086/321837
 This means that the morbidity and mortality from illnesses such as pneumonia and urinary tract infections will result in a huge social and economic burden on a country's health system. Furthermore, management for other types of illnesses may also be affected, because antibiotic treatment is crucial to the success of many types of surgery and of chemotherapy to treat cancers.3Harbarth, S., Balkhy, H. H., Goossens, H., Jarlier, V., Kluytmans, J., Laxminarayan, R., … Pittet, D. (2015). Antimicrobial resistance: One world, one fight. *Antimicrobial Resistance and Infection Control, 4*(1), 49. https://doi.org/10.1186/s13756-015-0091-2; O'Neill, *op. cit*. note 1.


All antimicrobial use promotes resistance. However, a key driver of avoidable AMR is antibiotic over‐prescription, that is, the clinically directed use of antibiotics for the treatment of conditions that would resolve satisfactorily without antibacterial therapy. AMR, once established, is potentially irreversible. Given the slow pace of discovery of newer antimicrobial agents,4Davies, J., & Davies, D. (2010). Origins and evolution of antibiotic resistance. *Microbiology and Molecular Biology Reviews, 74*(3), 417–433. https://doi.org/10.1128/mmbr.00016-10
 current treatment options can be considered a limited and non‐renewable resource. There is consequently an imperative to reduce antimicrobial prescribing to its bare minimum in an effort to eke out antibacterial efficacy for the greatest number of people, pending the development of new antibacterial classes or alternative approaches to infection treatment. Protecting this resource is of utmost importance to prevent a return to the ‘dark ages of medicine’ that preceded the discovery of penicillin in 1928.5Elhani, D. (2011). Does the emergence of antibiotic resistance announce the return of the dark ages? *Annales de Biologie Clinique, 69*(6), 637–646. https://doi.org/10.1684/abc.2011.0632; Platt, H. (1962). Moynihan: The education and training of the surgeon: Eleventh Moynihan lecture delivered at the University of Leeds on 25th May 1961. *Annals of the Royal College of Surgeons of England, 30*(4), 220. Given this importance, a consequentialist6Mill, J. S. (1998). *Utilitarianism*. Oxford: Oxford University Press. view might even go as far as prioritizing the protection of the existing antibiotic stock over the treatment of individual patients.7Foster, K. R., & Grundmann, H. (2006). Do we need to put society first? The potential for tragedy in antimicrobial resistance. *PLOS Medicine, 3*(2), e29. https://doi.org/10.1371/journal.pmed.0030029



Antibiotic prescribing can be conceptualized as a social dilemma, where the short‐term interests of individual patients and the long‐term interests of society are in conflict.8Baquero, F., & Campos, J. (2003). The tragedy of the commons in antimicrobial chemotherapy. *Revista Española De Quimioterapia, 16*(1), 11–13; Colman, A. M., Krockow, E. M., Chattoe‐Brown, E., & Tarrant, C. (2019). Medical prescribing and antibiotic resistance: A game‐theoretic analysis of a potentially catastrophic social dilemma. PloS one, *14*(4), e0215480. https://doi.org/10.1371/journal.pone.0215480 Foster & Grundmann, *op. cit*. note 7; Hollis, A., & Maybarduk, P. (2015). Antibiotic resistance is a tragedy of the commons that necessitates global cooperation. *The Journal of Law, Medicine & Ethics, 43*(3), 33–37. https://doi.org/10.1111/jlme.12272; O'Brien, K. S., Blumberg, S., Enanoria, W. T. A., Ackley, S., Sippl‐Swezey, N., & Lietman, T. M. (2014). Antibiotic use as a tragedy of the commons: A cross‐sectional survey. *Computational and Mathematical Methods in Medicine, 2014*(837929). https://doi.org/10.1155/2014/837929; Okeke, I. N. (2009). The tragedy of antimicrobial resistance: Achieving a recognition of necessity. *Current Science, 97*(11), 1564–1572; Porco, T. C., Gao, D., Scott, J. C., Shim, E., Enanoria, W. T., Galvani, A. P., & Lietman, T. M. (2012). When does overuse of antibiotics become a tragedy of the commons? *Plos One, 7*(12), e46505. Most antibiotic prescribing decisions are, at least initially, empirical, in that they are based on clinical judgement of the underlying causes of symptoms and the likely source of infection, rather than on microbiological confirmation of infective agents, and they are therefore fraught with uncertainties. From the perspective of the individual patient with symptoms suggestive of a bacterial infection but unknown pathogens, interests are to avoid severe illness or death. In most cases, these interests would be best served by the use of antibiotics, and broad‐spectrum antibiotics provide an easy and, in most cases, failsafe approach. This is because broad‐spectrum antibiotics target a wide range of pathogens. Even though they may cause side effects (e.g., clindamycin and quinolones may cause serious and even life‐threatening complications), many broad‐spectrum antibiotics can be a comparatively safe choice even if prescribed unnecessarily. From the perspective of wider society, a more conservative approach is preferred—avoiding unnecessary antibiotic use as far as possible, and using appropriate doses and durations of narrow‐spectrum antibiotics—in order to limit the drugs’ contribution to AMR.9Dyar, O. J., Obua, C., Chandy, S., Xiao, Y., Stålsby Lundborg, C., & Pulcini, C. (2016). Using antibiotics responsibly: Are we there yet? *Future Microbiology, 11*(8), 1057–1071. https://doi.org/10.2217/fmb-2016-0041
 Decisions about individual patients have societal consequences. AMR puts all at risk: even patients who have not had prior antimicrobial treatment can be infected by resistant micro‐organisms and suffer treatment failure as a consequence, and this will become increasingly problematic for patients in the future. Inequity of access to healthcare globally means that growing resistance will disproportionately affect poorer people, who will likely only have access to a more restricted range of antibiotic agents when they do receive treatment.

This social dilemma is complicated by the special role that doctors and healthcare staff play in providing access to antibiotic treatment. In most countries, antibiotic prescribing is a privilege reserved for clinicians, who act as antibiotic gatekeepers,10Broom, A., Plage, S., Broom, J., Kirby, E., & Adams, J. (2016). A qualitative study of hospital pharmacists and antibiotic governance: Negotiating interprofessional responsibilities, expertise and resource constraints. *BMC Health Services Research, 16*, 43–51. https://doi.org/10.1186/s12913-016-1290-0
 and patients need a doctor's prescription to gain legal access to antibiotics. This gatekeeping role developed in parallel with access to other drugs and was initially driven by the recognition that selecting the correct treatment, to maximize chances of recovery and minimize harm, required skilled analysis by trained doctors. The doctors' privileges were not primarily set up as a means of rationing access to care for the purposes of preserving resources. Given the growing problem of AMR, however, doctors have the difficult burden of balancing the interests of individual patients against the collective interests of future patients, which include preserving the resource of antimicrobial efficacy. Most doctors see it as their main duty to protect current patients, making the overuse of antibiotics a rational response under conditions of uncertainty. Over‐reliance on antibiotics can be reinforced by direct patient demands for antibiotics or by a doctor's perceptions of such demands.11Krockow, E. M., Colman, A. M., Chattoe‐Brown, E., Jenkins, D. R., Perera, N., Mehtar, S., & Tarrant, C. (2019). Balancing the risks to individual and society: A systematic review and synthesis of qualitative research on antibiotic prescribing behaviour in hospitals. *Journal of Hospital Infection,* 101(4), 428–439. https://doi.org/10.1016/j.jhin.2018.08.007; Little, P., Dorward, M., Warner, G., Stephens, K., Senior, J., & Moore, M. (2004). Importance of patient pressure and perceived pressure and perceived medical need for investigations, referral, and prescribing in primary care: Nested observational study. *BMJ, 328*(7437), 444–446. https://doi.org/10.1136/bmj.38013.644086.7c
 Importantly, a doctor's own interests can become tied to the overuse of antibiotics if they accrue financial and other benefits from antibiotic prescribing. This additional relationship function means that doctors ultimately hold significant accountability for the future evolution of resistant pathogens through over‐prescribing. By prioritizing the welfare of the patient in front of them, they threaten the welfare of other, future patients.

Ethical arguments make a strong case for the moral imperative to protect the rights of future, as yet unidentified, people.12Leibovici, L., Paul, M., & Ezra, O. (2012). Ethical dilemmas in antibiotic treatment. *Journal of Antimicrobial Chemotherapy, 67*(1), 12–16. https://doi.org/10.1093/jac/dkr425
 John Rawls’ principle of ‘justice between generations’,13Rawls, J. (1971). *A theory of justice*. Cambridge, MA: Belknap Press of Harvard University Press. clearly assumes moral equality between existing populations and future offspring, and the access of the latter to common resources, such as antibiotic efficacy. Rawls’ principle of justice would always stipulate a conservative and targeted prescribing approach in recognition of future patients.14Leibovici, *op. cit*. note 12. Doctors could be argued to hold responsibility for the rights of future patients, and to have a duty to decrease the harm to future patients even if this increases the risk to present patients. This could mean doctors having to make decisions that put current patients at slightly higher risk without their consent. It would also mean curtailing patients’ liberty to obtain an antibiotic even though patients may wish to do so to have the best chance of a positive outcome. Efforts to reduce antibiotic use may put doctors in a position of acting against patients’ preferences, which almost invariably lean towards the less restrictive use of broad‐spectrum antibiotics and therefore threaten the rights of future patients. In practice, however, limiting the autonomy of patients is ethically challenging, and the need for paternalistic prescribing might result in conflicted doctor–patient relationships, possibly affecting trust and respect.

Rawls’ concept of the ‘veil of ignorance’ provides a framework for assessing the ethics of doctors acting in line with a duty to future patients when this could conflict with the interests of current, individual patients.15Korobkin, R. (1998). Determining health care rights from behind a veil of ignorance. *University of Illinois Law Review, 1998*(3), 801; Rawls, *op. cit*. note 13. This concept considers how decision‐makers would choose if placed behind a veil of ignorance, which refers to an abstract choice scenario in which the decision‐makers are unaware of their own role (and own payoffs) in the situation. Rawls’ thought experiment would require decision‐makers to choose their preferred treatment approach, considering current patients with infection symptoms, future patients and non‐infected individuals, *independent of their personal roles*. Based on empirical results showing that decision‐makers behind the veil of ignorance typically prefer equitable healthcare solutions,16Andersson, F., & Lyttkens, C. H. (1999). Preferences for equity in health behind a veil of ignorance. *Health Economics, 8*(5), 369–378. https://doi.org/10.1002/(sici)1099-1050(199908)8:5<369::aid-hec456>3.0.co;2-q
 it could be argued that most people would generally agree on making appropriate efforts to preserve antibiotic efficacy for future patients through limiting antibiotic use with current patients.

Exceptions to this would be ethically justifiable in morally exceptional cases of extreme severity and urgency, where the death of an individual patient could easily be prevented.17Leibovici, *op. cit*. note 12. This moral reasoning is in line with Rawls’ idea of ‘minimising the worst outcome’,18Rawls, *op. cit*. note 13. and could also be termed an exceptional ‘Rule of Rescue’.19Jonsen, A. R. (1986). Bentham in a box: Technology assessment and health care allocation. *Law, Medicine and Health Care, 14*(3‐4), 172–174. https://doi.org/10.1111/j.1748-720x.1986.tb00974.x
 In the context of medical ethics, the Rule of Rescue has been previously considered when discussing resource allocation and deciding whether costly treatment options were justified for individual patients.20Cookson, R., McCabe, C., & Tsuchiya, A. (2008). Public healthcare resource allocation and the rule of rescue. *Journal of Medical Ethics, 34*(7), 540–544. https://doi.org/10.1136/jme.2007.021790; Schöne‐Seifert, B. (2009). The ‘rule of rescue’ in medical priority setting. *Rationality, Markets, and Morals, 4*, 421–430; Sheehan, M. (2007). Resources and the rule of rescue. *Journal of Applied Philosophy, 24*(4), 352–366. https://doi.org/10.1111/j.1468-5930.2007.00383.x
 An example in the context of antibiotic prescribing may be a patient with symptoms of severe sepsis where the fast administration of broad‐spectrum antibiotics could prevent almost certain death. The severity and immediacy of this outcome would mean that the rights of this patient would take precedence.21Leibovici, *op. cit*. note 12.


Recommendations for managing the ethical dilemma of antibiotic prescribing have pointed to the value of collective decisions, enacted through collectively agreed and implemented guidelines and decision support systems, rather than relying on individual doctors to shoulder the burden of balancing risks and benefits in each individual encounter.22Leibovici, *op. cit*. note 12. The importance of solidarity has also been emphasized, with a reorientation of the ethical focus towards considering collective interests and the fair distribution of risks and resources.23Littmann, J., & Viens, A. M. (2015). The ethical significance of antimicrobial resistance. *Public Health Ethics,* *8*(3), 209–224. https://doi.org/10.1093/phe/phv025



AMR is a global problem, not respecting country boundaries. International efforts are necessary to curb antibiotic use, but inequalities across high‐ and low‐income countries in access to resources mean that some countries bear a larger part of the burden.24Dyar, *op. cit*. note 9; Laxminarayan, R., Duse, A., Wattal, C., Zaidi, A., Wertheim, H., Sumpradit, N., … Cars, O. (2013). Antibiotic resistance – the need for global solutions. *The Lancet. Infectious Diseases, 13*(12), 1057–1097. https://doi.org/10.1016/s1473-3099(13)70318-9; Laxminarayan, R. (2016). The challenge of global antibiotic policy: Improving access and preventing excess. Retrieved from https://blogs.cdc.gov/global/2016/02/16/the-challenge-of-global-antibiotic-policy-improving-access-and-preventing-excess/
 Despite the growing literature on how to mitigate various aspects of the associated ethical dilemmas, and an increasing recognition of the imperative to act on a global scale,25Dyar, *op. cit*. note 9. little research has considered how the ethical dimensions that underpin decision‐making may be influenced by contextual factors, which are likely to vary across different regions, cultures or countries. Understanding and addressing specific factors that modify the prescribing dilemma for doctors across different nations is a crucial prerequisite to designing successful stewardship interventions or effective policies. Prescribing contexts are likely to differ most between countries with different socio‐economic backgrounds and different cultures. Previous literature has highlighted the fact that rich, industrialized nations continue to overconsume antibiotics. This is also the case in poorer nations, some of which are characterized by legal or illegal over‐the‐counter sales of antibiotics but which at the same time do not have sufficient access to high‐quality antibiotics or to more expensive second‐line treatments.26Laxminarayan et al. (2013), *op. cit*. note 24. Consequently, insufficient dosing is common in lower‐income countries, and this can also contribute to AMR.27Selgelid, M. (2007). Ethics and drug resistance. *Bioethics, 21*(4), 218–229. https://doi.org/10.1111/j.1467-8519.2006.00542.x
 Aspects of national culture,28Hofstede Insights. *Country comparison tool*. Retrieved from https://www.hofstede-insights.com/ [Accessed 29 Jan, 2019]. particularly uncertainty avoidance, hierarchy and masculinity, have also been found to be associated with levels of antibiotic consumption.29Borg, M. A. (2012). National cultural dimensions as drivers of inappropriate ambulatory care consumption of antibiotics in Europe and their relevance to awareness campaigns. *Journal of Antimicrobial Chemotherapy, 67*(3), 763–767. https://doi.org/10.1093/jac/dkr541; Deschepper, R., Grigoryan, L., Lundborg, C. S., Hofstede, G., Cohen, J., Van Der Kelen, G., … Haaijer‐Ruskamp, F. M. (2008). Are cultural dimensions relevant for explaining cross‐national differences in antibiotic use in Europe? *BMC Health Services Research, 8*(1), 123. https://doi.org/10.1186/1472-6963-8-123; Touboul‐Lundgren, P., Jensen, S., Drai, J., & Lindbæk, M. (2015). Identification of cultural determinants of antibiotic use cited in primary care in Europe: A mixed research synthesis study of integrated design ‘Culture is all around us’. *BMC Public Health, 15*(1), 908. https://doi.org/10.1186/s12889-015-2254-8
 Other aspects of cultural orientation, including individual versus collectivist orientation, and long‐ versus short‐term orientation, may also impact on how doctors weight the welfare of current and future patients, and their willingness to engage in collective endeavours to maintain antimicrobial efficacy.

The present article aims to explore how national context can shape the prominence of different dimensions of the ethical dilemmas in antibiotic prescribing decisions internationally, and receptiveness to solutions based on collective action and solidarity. This will be done by comparing three example countries, varying in their economic status, health system organization and delivery, cultural orientation, and geographical location: South Africa, Sri Lanka and the United Kingdom. We will focus on prescribing for acute medical patients in secondary care, because the hospital context is characterized by more complicated or serious cases of bacterial infections, which are associated with higher patient risks (notably fatal sepsis) and higher levels of treatment uncertainty, both of which sharpen the dilemma outlined above. The country analyses will consider national culture and the unique national environments of secondary‐care prescribing, and outline the key health policies relating to antibiotic use. This analysis is informed by input from expert collaborators (national experts in infection control and AMR from each of the three countries, who were collaborating on a funded project with the authors), and by visits by the two authors to seven hospitals (one public and one private hospital in South Africa; two public and one private hospital in Sri Lanka; two public hospitals in the U.K.) involving observations and discussions with local stakeholders. After the descriptions of national context, we will map the contextual factors against dimensions of the ethical dilemma. We will conclude with reflections of the impact of cultural context on the prescribing dilemma and the implications of this for the approach to designing stewardship interventions.

## 2 CONTEXTUAL FACTORS AFFECTING ANTIBIOTIC PRESCRIBING INTERNATIONALLY

This section will analyse contextual factors that may be associated with antibiotic prescribing dilemmas in South Africa, Sri Lanka and the U.K., outlining the general healthcare context and relevant national policies.

### 2.1 South Africa

South Africa is currently classified as a middle‐income country, with a gross domestic product per capita of 12,246 USD in 2016.30International Monetary Fund. (2018). *IMF data mapper*. Retrieved from http://www.imf.org/external/datamapper/PPPPC@WEO/OEMDC/ADVEC/WEOWORLD [Accessed 1 Jun, 2018]; The Global Economy. *Compare countries using data from official sources*. Retrieved from https://www.theglobaleconomy.com/compare-countries/
 It is characterized as a hierarchical, individualistic society, with an emphasis on competition and performance.31Hofstede Insights, *op. cit*. note 28. Healthcare inequalities are amongst the largest in the world,32Coovadia, H., Jewkes, R., Barron, P., Sanders, D., & McIntyre, D. (2009). Health in South Africa: The health and health system of South Africa: Historical roots of current public health challenges. *The Lancet, 374*(9692), 817. https://doi.org/10.1016/s0140-6736(09)60951-x
 and a big divide exists between public and private hospital care.33Schellack, N., Benjamin, D., Brink, A., Duse, A., Faure, K., Goff, D., … Essack. S. (2017). A situational analysis of current antimicrobial governance, regulation, and utilization in South Africa. *International Journal of Infectious Diseases, 64*, 100–106. https://doi.org/10.1016/j.ijid.2017.09.002
 Particular challenges, rooted in social and racial disparities, are posed by the high rates of HIV and tuberculosis (often present as co‐infections), which bind healthcare resources and potentially distract from other challenges.34Daftary, A., & Padayatchi, N. (2012). Social constraints to TB/HIV healthcare: Accounts from coinfected patients in South Africa. *AIDS Care, 24*(12), 1480–1486. https://doi.org/10.1080/09540121.2012.672719; Bogart, L. M., Chetty, S., Giddy, J., Sypek, A., Sticklor, L., Walensky, R. P., … Bassett. I. V. (2013). Barriers to care among people living with HIV in South Africa: Contrasts between patient and healthcare provider perspectives. *AIDS Care, 25*(7), 843–853. https://doi.org/10.1080/09540121.2012.729808; Karim, S. S. A, Churchyard, G. J., Karim, Q. A., & Lawn, S. D. (2009). Health in South Africa: HIV infection and Tuberculosis in South Africa: An urgent need to escalate the public health response. *The Lancet, 374*(9693), 921. https://doi.org/10.1016/s0140-6736(09)60916-8
 Tuberculosis infections are also increasingly becoming drug‐resistant, thus presenting a serious public health threat.35Andrews, J. A., Shah, N. S., Gandhi, N., Moll, T., & Friedland, G. (2007). Multidrug‐resistant and extensively drug‐resistant tuberculosis: Implications for the HIV epidemic and antiretroviral therapy rollout in South Africa. *The Journal of Infectious Diseases, 196*(3), S490. https://doi.org/10.1086/521121; Klopper, M., Warren, R. M., Hayes, C., van Pittius, N. C. G., Streicher, E. M., Muller, B., … Trollip, A. P. (2013). Emergence and spread of extensively and totally drug‐resistant Tuberculosis, South Africa. *Emerging Infectious Diseases, 19*(3), 449. https://doi.org/10.3201/eid1903.120246
 Antibiotic prescribing has been on the rise for many years, and this rise is disproportionately higher than in many other countries worldwide.36Meyer, J. C., Summers, R. S., & Möller, H. (2001). Randomized, controlled trial of prescribing training in a South African province. *Medical Education, 35*(9), 833–840. https://doi.org/10.1046/j.1365-2923.2001.01000.x; Paruk, F., Richards, G., Scribante, J., Bhagwanjee, S., Mer, M., & Perrie, H. (2012). Antibiotic prescription practices and their relationship to outcome in South African intensive care units: Findings of the prevalence of infection in South African intensive care units study. *South African Medical Journal, 102*(7), 613; Van Boeckel, T., Gandra, S., Ashok, A., Caudron, Q., Grenfell, B. T., Levin, S. A., & Laxminarayan, R. (2014). Global antibiotic consumption 2000 to 2010: An analysis of national pharmaceutical sales data. *Lancet Infectious Diseases, 14*(8), 742–750. https://doi.org/10.1016/s1473-3099(14)70780-7
 Consequently, AMR is a large and increasingly visible medical problem,37Bell, J. M. (2002). High prevalence of oxacillin‐resistant staphylococcus aureus isolates from hospitalized patients in Asia‐Pacific and South Africa: Results from SENTRY antimicrobial surveillance program, 1998–1999. *Antimicrobial Agents and Chemotherapy, 46*(3), 879–881. https://doi.org/10.1128/aac.46.3.880-882.2002; Liebowitz, L. D., Slabbert, M., & Huisamen, A. (2003). National surveillance programme on susceptibility patterns of respiratory pathogens in South Africa: Moxifloxacin compared with eight other antimicrobial agents*. Journal of Clinical Pathology, 56*(5), 344–347. https://doi.org/10.1136/jcp.56.5.344; Shittu, A. O., & Lin, J. (2006). Antimicrobial susceptibility patterns and characterization of clinical isolates of staphylococcus aureus in KwaZulu‐Natal Province, South Africa. *BMC Infectious Diseases, 6*(1), 125. https://doi.org/10.1186/1471-2334-6-125
 and one that contributes to high rates of hospital‐acquired infections.

Since 2012, the South African Antibiotic Stewardship Programme (SAASP), a multidisciplinary expert group, has been working to implement antibiotic stewardship programmes across primary and secondary care.38Schellack et al., *op. cit*. note 33. Their activities have been supported by South Africa's National Department of Health through the publication of a national strategy document in 2014, which defined a number of objectives, including the promotion of appropriate antibiotic use.39South Africa National Department of Health. (2014). *Antimicrobial resistance: National strategy framework 2014‐2024*. Retrieved from https://www.health-e.org.za/wp-content/uploads/2015/09/Antimicrobial-Resistance-National-Strategy-Framework-2014-2024.pdf
 The medical use of antibiotics in South Africa legally requires prescription. Currently, the most commonly prescribed antibiotic class is the broad‐spectrum penicillin oral class, which is produced nationally at comparatively low cost.40Schellack et al., *op. cit*. note 33 National guidelines for antibiotic prescribing exist in South Africa and are available electronically, but they do not apply to the private sector, where prescribing is based largely on the clinical evaluation of the practitioner in charge,41Chunnilall, D., Peer, A., Naidoo, I., & Essack, S. (2015). An evaluation of antibiotic prescribing patterns in adult intensive care units in a private hospital in KwaZulu‐Natal. *Southern African Journal of Infectious Diseases, 30*(1), 17–22. https://doi.org/10.1080/23120053.2015.1103956
 although there may be some carry over because most doctors working in the private sector also work in the public sector.

### 2.2 Sri Lanka

Sri Lanka is a middle‐income country in South Asia, with a similar gross domestic product per capita (11,639 USD, measured in 2016) to South Africa.42International Monetary Fund, *op. cit*. note 30; The Global Economy, *op. cit*. note 30. Sri Lanka has a hierarchical but collectively oriented culture with an emphasis on compromise, negotiation and self‐restraint.43Hofstede Insights, *op. cit*. note 28. Sri Lanka provides free hospital care, but in addition to this public healthcare system, many private hospitals exist. The differences between public and private hospitals are large,44Rannan‐Eliya, R. P., Wijemanne, N., Liyanage, I. K., Dalpatadu, S., de Alwis, S., Amarasinghe, S., & Shanthikumar, S. (2015). Quality of inpatient care in public and private hospitals in Sri Lanka. *Health Policy and Planning, 30*, i58. https://doi.org/10.1093/heapol/czu062
 and the nature of public provision differs in rural and urban settings based on varying resource levels.45Fernando, D. (2000). Health care systems in transition III. Sri Lanka, part I. An overview of Sri Lanka's health care system. *Journal of Public Health Medicine, 22*(1), 14–20. https://doi.org/10.1093/pubmed/22.1.14
 There is no public service for primary care, and the high fees of general practitioners as well as the limited opening hours of practices often result in delayed presentations of critically ill patients at hospital. A particularly prominent problem in Sri Lanka is that some patients are reluctant to access healthcare because of the economic consequences of being hospitalized, most notably being unable to work and support their families.46Tillekeratne, L. G., Bodinayake, C. K., Dabrera, T., Nagahawatte, A., Arachchi, W. K., Sooriyaarachchi, A., … Woods, C. W. (2017). Antibiotic overuse for acute respiratory tract infections in Sri Lanka: A qualitative study of outpatients and their physicians. *BMC Family Practice, 18*(37), 1–10. https://doi.org/10.1186/s12875-017-0619-z
 While antibiotics cannot be purchased legally in pharmacies without prescription, many pharmacies continue to dispense antibiotics to patients over the counter,47Wolffers, I. (1987). Drug information and sale practices in some pharmacies of Colombo, Sri Lanka. Social *Science & Medicine, 25*(3), 319–321. https://doi.org/10.1016/0277-9536(87)90234-6
 as is common in many other low‐ and middle‐income countries.48Morgan, D. J., Okeke, I. N., Laxminarayan, R., Perencevich, E. N., & Weisenberg, S. (2011). Non‐prescription antimicrobial use worldwide: A systematic review. *The Lancet Infectious Diseases, 11*(9), 692–701. https://doi.org/10.1016/s1473-3099(11)70054-8
 In fact, people frequently stock and keep antibiotics at home for self‐medication; this is a problem worldwide, but more so in countries where antibiotics are more freely available off prescription.

The first national guidelines for antibiotic prescribing were issued in 2016 by the Sri Lanka College of Microbiologists. The guidelines give recommendations on antibiotic treatment choices, but have only a limited focus on stewardship principles.49Sri Lanka College of Microbiologists. (2016). *Empirical and prophylactic use of antimicrobials: National guidelines 2016*. Retrieved from http://slmicrobiology.net/download/National-Antibiotic-Guidelines-2016-Web.pdf
 In addition, coordinated national initiatives exist to promote engagement in antibiotic stewardship, including a directive by the Ministry of Health,50Sri Lanka Ministry of Health, Nutrition & Indigenous Medicine. (2016). *Introduction of authorization of prescribing ‘red light’ antibiotics*. Retrieved from https://www.health.gov.lk/CMS/cmsmoh1/viewcircular.php?cno=01-56/2016&med=english
 which was recently issued to all public hospitals, identifying a number of ‘red‐light antibiotics’ for which prior microbiologist approval should be sought.51Sri Lanka College of Micobiologists. (2017). *Update on activities for combating antimicrobial resistance (AMR) in Sri Lanka ‐ April 2017*. Retrieved from http://slmicrobiology.net/update-on-activities-for-combating-antimicrobial-resistance-amr-in-sri-lanka-april-2017/



### 2.3 United Kingdom

The U.K. is a high‐income country with a GDP per capita of 39,254 USD (in 2016).52The Global Economy, *op. cit*. note 30. The U.K. culture is relatively non‐hierarchical, but individualistic and ‘indulgent’.53Hofstede Insights, *op. cit*. note 28. The majority of healthcare is delivered through the publicly funded National Health Service (NHS). With regard to the U.K.'s healthcare system, a recent OECD (Organisation for Economic Co‐operation and Development) report stated that, despite large British investments in healthcare quality improvement and a drive for innovation, the U.K. system achieves only average levels of care.54OECD. (2016). *OECD reviews of health care quality: United Kingdom 2016: Raising standards*. Paris, France: OECD Publishing. https://doi.org/10.1787/9789264239487-en
 Comparing the U.K. to other European nations with similar development status, antibiotic prescribing levels are in the mid‐range,55European Centre for Disease Prevention and Control. (2017). *Summary of the latest data on antibiotic consumption in the European Union*. Retrieved from https://ecdc.europa.eu/en/publications-data/summary-latest-data-antibiotic-consumption-eu-2017
 and an increase in antibiotic use has been observed for the secondary‐care sector despite overall hospital admissions decreasing.56Public and International Health Directorate/ Health Protection and Emergency Response Division/, HPP/ 10200. (2016). *DH UK 5 year antimicrobial resistance (AMR) strategy 2013–2018 – annual progress report, 2015*. Retrieved from https://www.gov.uk/government/uploads/system/uploads/attachment_data/file/553496/2nd_UK_AMR_annual_report.pdf



The topic of AMR has been on the U.K. political agenda for almost 20 years, with the Department of Health running antibiotic awareness campaigns every year since 1999 to educate both healthcare staff and the general public about the appropriate use of antibiotics.57Ashiru‐Oredope, D., Sharland, M., Charani, E., McNulty, C., & Cooke, J. (2012). Improving the quality of antibiotic prescribing in the NHS by developing a new antimicrobial stewardship programme: Start smart – then focus. *The Journal of Antimicrobial Chemotherapy, 67*(1), i63. https://doi.org/10.1093/jac/dks202
 Also, since 2001, the U.K. has been committed to collecting data on antibiotic use, which is fed into annual analyses and reports at the European Union (EU) level.58Gyssens, I. C. (2008). All EU hands to the EU pumps: The science academies of Europe (EASAC) recommend strong support of research to tackle antibacterial resistance. *Clinical Microbiology and Infection, 14*(10), 889–891. https://doi.org/10.1111/j.1469-0691.2008.02067.x
 Starting with the development of an initial antimicrobial stewardship package (High Impact Intervention) in 2009 by the Department of Health, more comprehensive national guidelines were released in 2011. With the title ‘Start Smart—Then Focus’, these guidelines combine an emphasis on the initial decisions to start antibiotics, including ensuring prompt administration of antibiotics in the case of sepsis or life‐threatening infections, with a recommendation to review, revise and stop antibiotic treatment as necessary.59Ashiru‐Oredope et al., *op. cit*. note 57.


The simultaneous emphasis on both sepsis prevention and antibiotic stewardship is also reflected in CQUINs (Commissioning for Quality and Innovation National goals), which aim to improve the response to sepsis as well as the reduction of MRSA (Methicillin‐resistant Staphylococcus aureus) and CDiff (Clostridium difficile) infections, thus recognizing the relationship and potential conflict in addressing these two themes.

## 3 IMPACT ON ETHICAL DECISION‐MAKING

Following the description of contextual factors in three countries varying in culture, economic development and health systems, this section will discuss the impact of these factors on the ethical dilemma outlined at the beginning of this article. We will consider four dimensions of the ethical dilemma against the country profiles from the previous section.

### 3.1 Visibility and moral equality of future generations

In Section 1, we argued that justice across generations, which rests on the assumed equality of current and future generations of identical moral status, is a key ethical imperative for antimicrobial stewardship. Out of the three countries examined, this imperative is most pronounced and visible in South Africa, where resistance levels are already very high and many patients suffer from medical complications as a result of AMR. Owing to this visibility, the temporal distance between current and future patients is blurred, potentially meaning that the recognition of the need to protect the rights to medical care for future generations of patients who may suffer the consequences of widespread AMR is more evident. Hence, even though antibiotic prescribing levels have been high in South Africa over recent years, doctors may now be forced to act on the increasingly visible consequences, which promote a ‘recognition of necessity’60Okeke, *op. cit*. note 8. for action to preserve antibiotic efficacy.

In contrast with the South African situation, little awareness exists amongst doctors in Sri Lanka about the problem of AMR.61Tillekeratne et al., *op. cit*. note 46; Tillekeratne, L. G., Bodinayake, C. K., Nagahawatte, A., Vidanagama, D., Devasiri, V., Arachchi, W. K., … Woods, C. W. (2015). Use of rapid influenza testing to reduce antibiotic prescriptions among outpatients with influenza‐like illness in southern Sri Lanka. *American Journal of Tropical Medicine and Hygiene, 93*(5), 1031–1037. https://doi.org/10.4269/ajtmh.15-0269
 There is a lack of information about local resistance patterns, and many doctors treating acute medical patients show little concern about the health threat posed by AMR. In the public hospital sector, this lack of interest may be due to more pressing problems, including limited bed space and understaffing.62Tillekeratne et al., *op. cit*. note 46. Furthermore, the choice of antibiotics is often severely limited based on treatment costs.

This situation is further complicated by the fact that many doctors distrust the efficacy of cheaper, unbranded antibiotics, which are manufactured locally rather than imported from international pharmaceutical companies. This distrust matches doctors’ attitudes in the neighbouring country India, where antibiotics vary significantly in price,63Patel, D., Thiyagu, R., Suruyivelrayan, N., Patel, H., & Pandey, S. (2009). Price variability among the oral antibiotics available in a south Indian tertiary care hospital. *Journal of Clinical and Diagnostic Research, 3*, 1871–1875. and doctors typically only trust the more costly, imported brands.64Patel, V., Vaidya, R., Naik, D., & Borker, P. (2005). Irrational drug use in India: A prescription survey from Goa. *Journal of Postgraduate Medicine, 51*(1), 9–12. To pre‐empt any negative patient outcomes based on the limited efficacy of cheap antibiotic drugs, many hospital doctors prescribe higher doses of these antibiotics, or multiple antibiotic types. In addition, studies found that poor hygiene and sanitary conditions in South Asian countries often resulted in the preventative use of antibiotics, for example to protect frail patients in a contaminated environment.65Nguyễn, M. H., Gammeltoft, T., Christoffersen, S. V., Trần, T. T., & Rasch, V. (2009). Reproductive tract infections in northern Vietnam: Health providers’ diagnostic dilemmas. *Women & Health, 49*(2‐3), 229–245. https://doi.org/10.1080/03630240902963630; Om, C., Daily, F., Vlieghe, E., McLaughlin, J. C., & McLaws, M. (2016). ‘If it's a broad spectrum, it can shoot better’: Inappropriate antibiotic prescribing in Cambodia. *Antimicrobial Resistance & Infection Control, 5*(1), 1–8. https://doi.org/10.1186/s13756-016-0159-7
 These practices can result in antibiotic overuse and an accelerated increase of AMR; justice across generations matters less to doctors in these demanding circumstances than dealing with immediate risks.

British doctors also experience a lower visibility of AMR than doctors in South Africa, and currently have few negative patient outcomes owing to multidrug‐resistant infections. Many lack concern for the problem of AMR and have little insight into local levels of resistance, considering it to be a vague, hypothetical threat, loosely coupled to their actions.66Broom, J. K., Broom, A. F., Kirby, E. R., Gibson, A. F., & Jeffrey, J. P. (2017). Clinical and social barriers to antimicrobial stewardship in pulmonary medicine: A qualitative study. *American Journal of Infection Control, 37*(8), 1–5. https://doi.org/10.1016/s0196-6553(09)00726-3
 Instead, most perceive a very strong duty of care towards individual patients. Inexperienced frontline doctors are frequently risk‐averse and fear criticism from senior colleagues for failing to treat a patient.67Charani, E., Edwards, R., Sevdalis, N., Alexandrou, B., Sibley, E., Mullett, D., … Holmes, A.. (2011). Behavior change strategies to influence antimicrobial prescribing in acute care: A systematic review. *Clinical Infectious Diseases, 53*(7), 651–662. https://doi.org/10.1093/cid/cir445; Rawson, T. M., Charani, E., Moore, L. S. P., Hernandez, B., Castro‐Sánchez, E., Herrero, P., … Holmes, A. H. (2016). Mapping the decision pathways of acute infection management in secondary care among UK medical physicians: A qualitative study. *BMC Medicine, 14*(1) https://doi.org/10.1186/s12916-016-0751-y
 This risk‐aversion is partly due to the overriding focus on prevention of mortality from sepsis and other infections, which is politically and socially driven.68Eyer, M. M., Läng, M., Aujesky, D., & Marschall, J. (2016). Overtreatment of asymptomatic bacteriuria: A qualitative study. *Journal of Hospital Infection, 93*(3), 297–303. https://doi.org/10.1016/j.jhin.2016.04.007
 Additional factors that prevent more targeted prescribing, and changing or de‐escalating antibiotics, are time pressures and high demands on junior doctors. Especially during sparsely staffed night shifts, it is easier to cover patients with high doses of broad‐spectrum antibiotics than to invest time and cognitive effort in determining a more targeted treatment option.69Broom, J., Broom, A., Plage, S., Adams, K., & Post, J. J. (2016). Barriers to uptake of antimicrobial advice in a UK hospital: A qualitative study. *Journal of Hospital Infection, 93*(4), 418–422. doi://dx.doi.org/10.1016/j.jhin.2016.03.011; Broom et al., *op. cit*. note 66. Individual perceptions of risks and benefits associated with broad‐spectrum antibiotic use are discussed in more detail in a recent literature review.70Krockow et al., *op. cit*. note 11.


These contextual factors in the U.K. are at odds with the imperative for equality across generations. First, the current threat of AMR is masked, allowing doctors to put aside concerns about the extent to which AMR will actually apply to future generations. Secondly, these factors indicate a clear prioritization of current patients, which is in stark contrast with the right of future societies to the same antibiotic treatment options as available to previous generations.

### 3.2 Rule of Rescue

Another important dimension of the ethical dilemma is represented by the Rule of Rescue. In Section 1, we argued that extreme cases of urgency can justify extraordinary actions of rescue. For example, symptoms of severe sepsis may justify immediate prescriptions of broad‐spectrum antibiotics. The prevailing importance of a doctor's concern for immediate patient needs was supported by results from a study that investigated the allocation of limited intensive care unit (ICU) beds in U.K. hospitals. The study's investigators concluded that the doctor's perception of Rule of Rescue ‘represents a substantial and persistent barrier to the efficient allocation of scarce resources’.71Kohn, R., Rubenfeld, G., Levy, M., Ubel, P., & Halpern, S. (2011). Rule of rescue or the good of the many? An analysis of physicians’ and nurses’ preferences for allocating ICU beds. *Intensive Care Medicine, 37*(7), 1210–1217. https://doi.org/10.1007/s00134-011-2257-6



The Rule of Rescue received some attention from the U.K.'s National Institute for Health and Clinical Excellence (NICE), which charged the Citizens Council with the preparation of an ethical report on the issue. The Citizens Council is a panel of British lay people chosen to represent the current demographic of the U.K. The panel's purpose is to discuss controversial health topics and formulate recommendations for NICE guidance based on their public perspective. In 2006, the Citizens Council discussed various aspects of the Rule of Rescue, but their final report remained non‐specific in its conclusions.72
*Rule of Rescue: Citizens council reports no. 6*. (2006). Retrieved from https://www.nice.org.uk/Media/Default/Get-involved/Citizens-Council/Reports/CCReport06RuleOfRescue.pdf



Despite this official lack of clarity surrounding the Rule of Rescue, it appears to be deeply embedded within the U.K.'s national guidelines on antibiotics and may present a significant barrier in the context of appropriate allocation of antibiotic treatment. The Start Smart, then Focus guidance emphasizes the importance of immediate action to prevent mortality from sepsis. While it is also designed to optimize antibiotic use, the prominent focus in hospitals on rescue of patients with sepsis may distract from considerations of long‐term consequences. Because sepsis has ambiguous symptoms and the risk of failing to treat are severe, over‐diagnosis is common, and many patients are mistakenly classified as septic and given antibiotics: the Rule of Rescue is sometimes invoked inappropriately. In addition, while the guideline's first part (‘start smart’) has readily been implemented across hospitals, studies have shown that many institutions fail to consistently follow the guideline's second part (‘then focus’)73Llewelyn, M. J., Hand, K., Hopkins, S., & Walker, A. S. (2015) Antibiotic policies in acute English NHS trusts: implementation of ‘Start Smart—Then Focus’ and relationship with *Clostridium difficile* infection rates. *Journal of Antimicrobial Chemotherapy, 70*(4), 1230–1235; Ashiru‐Oredope, A., Budd, E. L., Battacharya, A., Din, N., McNulty, C. A. M., Micallef, C., et al. (2016) Implementation of antimicrobial stewardship interventions recommended by national toolkits in primary and secondary healthcare sectors in England: TARGET and Start Smart Then Focus, *Journal of Antimicrobial Chemotherapy*, *71*(5), 1408–1414., which involves narrowing down the initial drug choice, and thus revising the treatment in order to adopt a more conservative approach. The necessary treatment review is often delayed or does not happen at all. In this case, the focus on the Rule of Rescue means that once the initial rescue has been performed, the subsequent review and revision of the prescribing decision is given lower priority.

A problematic but different challenge to the ‘Rule of Rescue’ became evident in the context of Sri Lanka. Whereas severe sepsis is often over‐diagnosed in the U.K., and urgency of treatment is frequently overestimated, Sri Lankan hospitals are characterized by a genuinely higher proportion of emergency cases that require more drastic action. As pointed out in the previous section, many patients in Sri Lanka present to hospital very late and only once the infection has reached a dangerous stage.74Tillekeratne et al., *op. cit*. note 46. The lack of public‐sector primary care as well as patients’ worries about missing work and losing income mean that by the time patients are admitted to hospital, they may indeed require ‘rescue’ by administration of broad‐spectrum antibiotics, limiting the ability of hospital doctors to make more conservative prescribing decisions. In addition, because of the lack of adequate onsite microbiology laboratories in many public hospitals, the clinical certainty is even lower than in more developed countries, which makes a focus on reviewing and switching from broad‐spectrum to narrower‐spectrum antibiotics once a patient has been ‘rescued’ with broad‐spectrum antibiotics more difficult to achieve. The prescribing logic is similar in the public sector in South Africa.

### 3.3 Prescribing autonomy and conflicts of interest

Contexts in which doctors retain full decision autonomy over antibiotic prescribing, but where significant conflicts of interest exist that incentivize antibiotic prescribing, can become problematic. A particular example appears to be Sri Lanka's private healthcare sector. The incentive structure for hospitals in the private sector, and for the doctors who work within them, results in a privileging of current individual patient outcomes, both clinical and experience‐based, over the interests of generations to follow. The sector is characterized by high levels of competition between hospitals to attract patients, a strong business orientation, and significant investment in marketing. In Sri Lanka's private hospitals, most doctors are employees of the public sector hospitals but also work in private hospitals to augment their relatively low public sector salaries. Doctors are dependent on their extra private practice income, which in turn depends on a continuous influx of patients. As such, doctors typically aim to please their private patients. Widespread patient beliefs about antibiotics as strong and powerful drugs, and as having an almost mythical status, mean that patients often demand and expect to receive antibiotics.75Tillekeratne et al., *op. cit*. note 46. Even if private consultants believe in the necessity to preserve antibiotic efficacy, they are aware that patients can choose to ‘shop around’ for other doctors until obtaining their preferred prescriptions. This practice, which has also been observed in other South Asian countries,76Radyowijati, A., & Haak, H. (2003). Improving antibiotic use in low‐income countries: An overview of evidence on determinants. *Social Science & Medicine, 57*(4), 733–44. https://doi.org/10.1016/s0277-9536(02)00422-7
 results in doctors being disempowered to act to protect the collective interest. Furthermore, in the hospitals we visited, the insurance reimbursement schemes left doctors with discretion to prescribe excessively to meet patient demand without scrutiny. With no incentive or pressure to rationalize antibiotic prescribing, doctors rarely curb or refine their treatment strategy.

Doctors working in private healthcare in South Africa face the same pressures to satisfy patient demand as those apparent in Sri Lanka, but, in contrast, we observed healthcare insurance reimbursement schemes in private hospitals that required detailed reporting of resource use, and that limited payments for antibiotic use. This incentive scheme resulted in tighter organizational monitoring and control of prescribing decisions, balancing out incentives to respond to patient demand. This is an example of a shift towards the interests of society driven by financial incentives as opposed to the moral reasoning of individual doctors; however, the same in the two cases.

As identified above, the use of collectively agreed guidelines provides a means of supporting doctors to make ethical decisions about restricting the use of antibiotics without patient consent—although whether or not doctors adhere to these guidelines is another matter, particularly where there are conflicts of interest. Insurance reimbursement schemes can act to support or undermine implementation of such guidelines by reinforcing or discouraging an individual prescriber's inclination to choose their treatment without consideration of collective interests.

### 3.4 Consensus on collective action

We have already established that collectively acceptable prescribing guidelines or decision support tools provide a way of overcoming ethical problems associated with representing the interests of future patients, but the introduction and implementation of such guidelines requires consensus, collaboration and collective monitoring.

South Africa appears to provide a national context where such consensus about the decision approach exists, to some degree, in the public sector. First, it has guidelines, which are binding for the public hospital sector.77South Africa National Department of Health, *op. cit*. note 39. Furthermore, the high level of AMR, which is observed on a regular basis, has resulted in a greater consensus about the need to target drug resistance and change previous prescriber behaviour.78Andrews et al., *op. cit*. note 35. Rather than conceptualizing AMR as an abstract challenge, doctors regard drug resistance as a practical problem complicating the treatment and management of HIV‐related infections, tuberculosis and the high rates of hospital‐acquired infections. As a result, many hospital doctors weigh up their prescribing choices carefully and consider possible treatment complications resulting from an increase in AMR. A national consensus is being invoked towards AMR by the work of the recently convened Ministerial Advisory Committee on AMR. The Committee is finalizing guidelines, which are to be published imminently. The use of laboratory results to guide antibiotic stewardship is being instituted with local sensitivity patterns being considered in some hospitals, and is being used to guide antibiotic prescribing policies in several provinces. Hence, the antibiotic stewardship programme is working towards a collective approach, which supports and informs doctors about the way to best use microbiology laboratories and pharmacists about monitoring the appropriate use of antibiotics. Despite this high visibility, antibiotic prescribing ultimately depends on the local enforcement of existing guidelines, but resources to support implementation and enforcement are unequally distributed across public and private hospitals. Even though antibiotic stewardship groups have been formed to review and optimize use of antibiotics across most South African hospitals, many public hospitals (especially in rural areas) have limited means to support these groups effectively. For example, public hospitals typically rely on pen‐and‐paper prescribing systems, which complicate the monitoring of antibiotic prescribing. Hence, audits of guideline adherence can be difficult.

Although antibiotic prescribing guidelines do not apply to the private healthcare sector in South Africa, and therefore doctors have higher levels of prescribing freedom,79Park et al., *op. cit*. note 36. the superior resource environment of private hospitals, including electronic record‐keeping systems, makes it easier to track and review antibiotic prescribing.80Schellack et al., *op. cit*. note 33.


In Sri Lanka, a strongly coordinated, consensus‐based approach to antimicrobial stewardship is led by the Sri Lankan College of Microbiologists, but only within the public sector. Guidelines are very rarely followed in private hospitals. Adherence to the guidelines is not, however, monitored or audited in either sector.81Wolffers, *op. cit*. note 47. In public hospitals, most antibiotic prescribing is undertaken by junior doctors, who typically require microbiology sign‐off to prescribe red‐light antibiotics. However, owing to the limited reviews of antibiotics and the generally passive role of nurses and pharmacists, stopping or de‐escalating antibiotic treatment can be a problem. Furthermore, many low‐resource hospitals are forced to prioritize economic considerations over concerns regarding AMR. Often, the choice of an antibiotic is dependent on the drug's cost and affordability to patients;82Ibid. this is particularly important if a prolonged course of antibiotic treatment is necessary.

The testing of samples in local microbiology laboratories is becoming more frequent in the public health sector, but the trust of doctors in local test results is limited as a result of a perceived lack of local expertise and poor hygiene conditions. In those cases where no laboratory facilities exist on‐site, samples need to be sent to larger laboratories, which leads to delays of test results and extended periods of empirical prescribing.83Tillekeratne et al., *op. cit*. note 49. On the whole, the existence of other pressing problems, and the higher levels of clinical uncertainty in Sri Lanka make it difficult to implement the collectively agreed antibiotic prescribing strategy in practice.

In the U.K., the national approach towards antibiotics is also much less defined than in South Africa, with antibiotic stewardship guidelines and initiatives potentially competing with other priorities, including sepsis prevention.84Broom et al., *op. cit*. note 71. Furthermore, individual hospital trusts typically develop their own sets of local prescribing guidelines to reflect regional resistance patterns. The hospitals retain authority to design their own types of antibiotic prescribing documents, restrictions and processes as they see fit.85Woodford, E. M., Wilson, K. A., & Marriott, J. F. (2004). Documentation of antibiotic prescribing controls in UK NHS hospitals. *The Journal of Antimicrobial Chemotherapy, 53*(4), 650–652. https://doi.org/10.1093/jac/dkh152
 In addition, hospitals differ in their restrictive policies. While some impose pre‐emptive policies such as restricting maximum doses or durations of antibiotics or mandating microbiology sign‐off for certain broad‐spectrum antibiotics, other hospitals have a stronger focus on post‐prescribing checks to refine antibiotic choice and duration by antimicrobial pharmacists or antibiotic stewardship teams. However, the frequently non‐binding guidelines and interventions leave room for interpretation by individual doctors and ways to circumvent the system.86LaRosa, L. A., Fishman, N. O., Lautenbach, E., Koppel, R. J., Morales, K. H., & Linkin, D. R. (2007). Evaluation of antimicrobial therapy orders circumventing an antimicrobial stewardship program: Investigating the strategy of ‘stealth dosing’*. Infection Control and Hospital Epidemiology, 28*(5), 551. The hospitals’ expert doctors (i.e., microbiologists or infectious diseases specialists) are typically in charge of the stewardship approach and responsible for delivering on national CQUIN targets. Within the context of hospital hierarchies, it is often unclear how much of the information on national targets filters down to frontline prescribing staff, who are mainly junior doctors. It is this pervasive lack of consensus that complicates the role of guidelines in supporting ethical decision‐making about antibiotic use the U.K.

## 4 SUMMARY AND CONCLUSIONS

We have described how various dimensions of the ethical dilemma of prescribing antibiotics may be forefronted or attenuated in different international settings, owing to cultural and structural differences in healthcare systems and healthcare provision. The extent to which AMR is a visible threat influences the orientation of doctors towards actions that preserve the collective interests in preserving antimicrobial efficacy for the future. In South Africa, unlike in the U.K. or Sri Lanka, doctors do not have to imagine the future generations whom they have a moral duty to protect from the consequences of AMR—they regularly see patients who are already suffering these consequences, particularly in the public sector. For these doctors, the distinction between the rights of current individuals and future generations is blurred. Also, their engagement in working towards the collective goal of conservation of antibiotic efficacy is likely to be higher, because it has immediate consequences for themselves and their patients. In public hospitals in Sri Lanka, limitations in microbiology testing lead to a lack of information about resistance levels. The ethical principal of justice for future generations is overshadowed by the Rule of Rescue, given the severe and urgent condition of many patients. In settings where antibiotic resistance is hidden, and the risk of patient mortality is widespread and high, the ethical principles of justice for future generations are more likely to be played down. Engaging doctors in collective efforts to preserve antimicrobial efficacy will require a ‘recognition of necessity’87Okeke, *op. cit*. note 8. through making clear the growing scale and immediacy of the problem. This needs to be balanced, particularly in low‐ and middle‐income countries, by supporting doctors to optimize their prescribing without significantly increasing immediate mortality risks. The approaches required will vary across settings and international contexts.

Sri Lanka has a collectively orientated culture, and this is reflected in the approach in the public sector to consensus‐based guidelines, supposed to be implemented consistently across the whole country. The national culture acts as a fertile ground for coordinated, society‐based approaches to antimicrobial stewardship. Problems arise, however, from a lack of infrastructure to support monitoring and auditing of practice to maintain this in line with collective goals. Furthermore, hierarchies across professional roles (e.g., doctors, nurses and microbiologists) act as barriers to an inclusive approach to working together in stewardship activities. Interdisciplinary involvement in guideline development and implementation, and the development of approaches for the collective monitoring of practice may help to support ethical decision‐making.

Patient choice and demand are significant considerations in the private healthcare settings of South Africa and Sri Lanka. Doctors are dependent on individual income and reputation, and organizations and individuals are often in direct competition with each other. The stark contrast between Sri Lanka's and South Africa's private sectors shows how economic incentives can substitute for morally and ethically based solutions, but that the systems for implementing these need to be designed to ensure that economic incentives and sanctions line up with collective goals for the conservation of antimicrobial efficacy.

In the U.K., levels of resistance are lower than in South Africa, and doctors lack feedback on resistance levels. Furthermore, the availability of alternative treatments (e.g., second‐ and third‐line antibiotics) means that treatment complications as a result of AMR are rare. Like South Africa and Sri Lanka, the U.K. also has national initiatives to promote antibiotic stewardship, but a history of inter‐organizational competition and a culture of local priority‐setting and planning has contributed to a lack of consensus and collaboration. The U.K. context also highlights how goals to reduce drug‐resistant infections can be crowded out by more immediate concerns about mortality from sepsis. Supporting U.K. doctors to make ethical decisions about antibiotic use that protect the interests of society may require efforts to make visible the problem of resistance. It may also necessitate national discussion of ethical principles to develop consensus on the prioritization of different interests under different circumstances.

AMR is a worldwide problem that can be effectively tackled only by concerted global action. In view of the gravity of this problem in the medium to long term, reforms in prescribing practices, no doubt slightly different in different countries, are required to avoid a catastrophic outcome. The problem may well be tractable, but a nuanced understanding of how the national and local context within which prescribing takes place shapes the nature of the dilemma is critical to inform the design of effective approaches.

## CONFLICT OF INTEREST

The authors declare no conflict of interest.

## FUNDING INFORMATION

This research was funded by the Global Challenges Research Fund—Grant No. ES/P004784/1 awarded by the Economic and Social Research Council (ESRC) on behalf of the Research Councils U.K. (RCUK).

